# Antihypertensive Effect of a Self-Microemulsifying System Obtained from an Ethanolic Extract of *Heliopsis longipes* Root in Spontaneously and L-NAME-Induced Hypertensive Rats

**DOI:** 10.3390/molecules30183711

**Published:** 2025-09-12

**Authors:** Dailenys Marrero-Morfa, Beatriz A. Luz-Martínez, Francisco J. Luna-Vázquez, Carlos T. Quirino-Barreda, Isela Rojas-Molina, Martín García-Servín, Pedro A. Vázquez-Landaverde, Victoria Ruiz-Castillo, César Ibarra-Alvarado, Alejandra Rojas-Molina

**Affiliations:** 1 Posgrado en Ciencias Químico Biológicas, Facultad de Química, Universidad Autónoma de Querétaro, Querétaro 76010, Mexico; dailenys.marrero@uaq.mx (D.M.-M.); bluzmartinez2@gmail.com (B.A.L.-M.); gruiz05@alumnos.uaq.mx (V.R.-C.); 2 Laboratorio de Investigación Química y Farmacológica de Productos Naturales, Facultad de Química, Universidad Autónoma de Querétaro, Querétaro 76010, Mexico; francisco.luna@uaq.mx (F.J.L.-V.); jjirojasmolina@gmail.com (I.R.-M.); 3 División de Ciencias Biológicas y de La Salud, Departamento de Sistemas Biológicos, Universidad Autónoma Metropolitana, Ciudad de México 04960, Mexico; cquirino@correo.xoc.uam.mx; 4 Laboratorio Universitario Bioterio, Instituto de Neurobiología, Universidad Nacional Autónoma de México, Querétaro 76230, Mexico; garciasm@unam.mx; 5 Centro de Investigación en Ciencia Aplicada y Tecnología Avanzada del Instituto Politécnico Nacional, Unidad Querétaro, Querétaro 76090, Mexico; pavazquez@ipn.mx

**Keywords:** affinin, antihypertensive effect, spontaneously hypertensive rats, endothelial reactivity, *Heliopsis longipes*, SMEDDS

## Abstract

Arterial hypertension is a major contributor to cardiovascular disease, the leading cause of death globally. Previously, our research group has demonstrated that both organic extracts from *Heliopsis longipes* roots and affinin—its principal bioactive compound—induce vasodilation and exert antihypertensive effects in L-NAME-induced hypertensive rats. However, the poor water solubility of these extracts limits their oral administration and dosing. To address this limitation, a self-microemulsifying drug delivery system (HL-SMDS) was developed from an ethanolic extract of *H. longipes* root to enhance its aqueous solubility and oral bioavailability. This study evaluated the antihypertensive efficacy of HL-SMDS in spontaneously hypertensive and L-NAME-induced hypertensive rat models, as well as its effects on endothelial reactivity. HL-SMDS significantly reduced systolic blood pressure in both models, demonstrating greater efficacy than the crude extract, likely due to improved solubility and systemic bioavailability of the active constituents. Moreover, HL-SMDS enhanced endothelial function in aortas from L-NAME-treated rats. These findings support the potential of HL-SMDS as a lipid-based phytopharmaceutical formulation that improves the oral bioavailability and antihypertensive effect of the ethanolic extract of *H. longipes* root. HL-SMDS offers a promising strategy for the development of phytopharmaceutical drugs to treat hypertension.

## 1. Introduction

Arterial hypertension is one of the most important factors contributing to the development of cardiovascular diseases, which are the main cause of death worldwide [1]. High blood pressure affects more than 1.2 billion people around the world and has become the most critical and expensive public health problem [2,3]. International guidelines suggest the use of a triple treatment; however, despite the use of this combination therapy, patients continue with high blood pressure values, which is a characteristic of resistant hypertension [4,5].

Blood pressure remains difficult to control with current antihypertensive medications [6]. Given the numerous adverse effects associated with these drugs—including electrolyte and metabolic abnormalities, dry cough, angioneurotic edema, anemia, renal failure, and peripheral edema, among others [7]—it is evident that continued research into new pharmacological targets and therapeutic agents is essential to achieve effective blood pressure control [6]. Within this framework, plants used in traditional medicine represent a valuable source of lead compounds for the development of more effective and safer drugs to treat hypertension and other cardiovascular diseases [8,9].

*Heliopsis longipes* is a botanical species endemic to Mexico that is used in Mexican traditional medicine to relieve toothache and as an antiparasitic and insecticide [10,11,12]. Affinin, also known as spilanthol, is the main alkamide present in *H. longipes* root, and it is considered responsible for the biological effects displayed by this plant. Extracts prepared from *H. longipes* root and pure affinin have shown different biological and pharmacological activities [13,14,15]. Our research group previously demonstrated the vasodilator effect of affinin and organic extracts of *H. longipes* root [11,16], and recently, we found that both the extracts and pure affinin produce an antihypertensive effect on L-NAME-induced hypertensive rats, which is mediated by the activation of CB1 cannabinoid receptors type 1 (CB1) and Transient Receptor Potential Ankyrin 1 (TRPA1) and Transient Receptor Potential Vanilloid 1 (TRPV1) channels [17]. However, the organic extracts obtained from the root of this species have poor water solubility, which makes them difficult to administer and to dose accurately. For this reason, our research group developed a self-microemulsifying system loaded with an ethanolic extract of *H. longipes* root (HL-SMDS) [18] in order to increase its aqueous solubility and enhance its bioavailability. HL-SMDS, which consisted of spherical droplets with a size of less than 100 nm, remained stable over time under extreme conditions. Furthermore, the release of affinin from HL-SMDS was significantly increased with respect to the release from the crude extract.

In this context, the present study aimed to evaluate whether oral administration of HL-SMDS reduces blood pressure in two widely employed rodent models of hypertension: the L-NAME-induced hypertension model and the spontaneously hypertensive rat (SHR) model. The L-NAME model, based on chronic inhibition of nitric oxide synthase, induces sustained hypertension through reduced endothelial NO production and reproduces several complications of human disease, including renal dysfunction, fibrosis, and hypertensive microangiopathy. This model is highly reproducible, technically simple, reversible with standard antihypertensive drugs, and extensively used, thereby supporting its validity for investigating vascular dysfunction [19]. In contrast, the SHR model constitutes a genetic model that closely resembles human essential hypertension, integrating vascular, renal, and neurohumoral mechanisms and, thus, enabling long-term evaluation of antihypertensive therapies [20]. In addition to assessing its antihypertensive activity, this study also investigated whether HL-SMDS was capable of improving endothelial reactivity, and a preliminary analysis of its potential cardioprotective effect was performed.

## 2. Results

### 2.1. GC-MS Analysis of the Ethanolic Extract from H. Longipes Root (EEH)

Gas Chromatography–Mass Spectrometry (GC-MS) analysis of EEH allowed the identification of a total of 27 low-molecular-weight volatile compounds. Among these, five were fatty acids or their derivatives, nine were terpenes (including mono-, sesqui-, and triterpenes), two were tocopherol derivatives, four were alkamides, two were phytosterols, two were phenylpropanoids, and three were alkanes (Table 1).

Based on relative abundance, EEH is mainly composed of alkamides, which account for 45.57% of the extract. Within this group, affinin is the most abundant compound (45.08%), followed by N-(2-methylbutyl)-(2*E*,6*Z*,8*E*)-decatrienamide (0.41%), 13-Docosenamide, and (*Z*)-(0.05%) and N-isobutyl-(6*Z*,8*E*)-decadienamide (0.03%). In addition to alkamides, the most representative compounds in the extract include stigmasterol (0.37%) and the triterpenes lup-20(29)-en-3-one (0.23%) and taraxasterol (0.21%).

### 2.2. Obtaining the Self-Microemulsifying System Loaded with the Quantified EEH

The optimal composition of the lipid system, which consisted of Transcutol^®^: 55%, Tween80^®^/ propylene glycol (PG)^®^: 10%, and Labrasol^®^: 35%, was loaded with the quantified EEH containing an average affinin content of 342 ± 0.3 µg/mg of extract. This system was characterized as previously described [18].

### 2.3. Determination of the Antihypertensive Effect of HL-SMDS in L-NAME-Induced Hypertensive Rats

**Figure 1:**
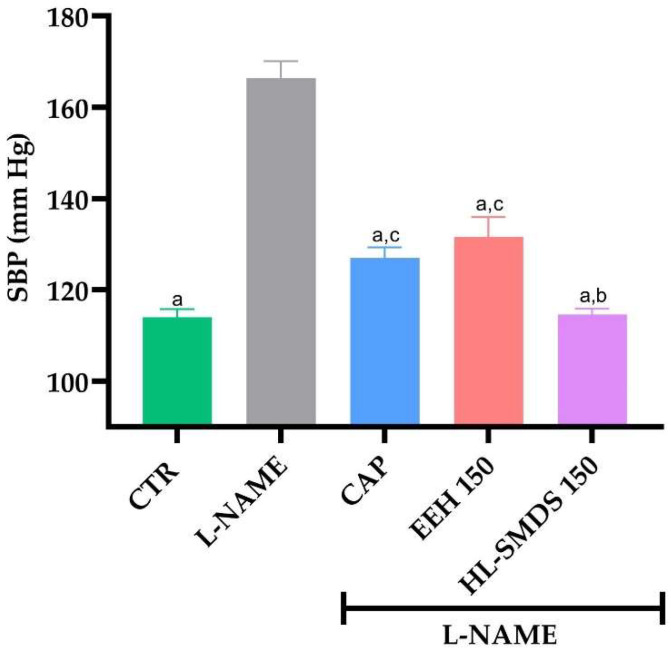
Effect of oral administration of EEH and HL-SMDS (150 mg/kg/day) over 4 days on the SBP of L-NAME-induced hypertensive rats. A dose of HL-SMDS corresponding to 150 mg/kg contains an approximate dose of affinin of 51 mg/kg. Captopril (CAP) (100 mg/kg/day) was used as a positive control. Values are the mean ± SEM (*n* = 6 rats for each group). ^a^ *p* < 0.05 vs. L-NAME, ^b^ *p* < 0.05 EEH 150 vs. HL-SMDS 150; ^c^ *p* < 0.05 vs. CTR using one-way ANOVA followed by Tukey’s post hoc test.

After 3 weeks of L-NAME administration, an increase in blood pressure in the rats of all four groups (L-NAME, Captopril, EEH 150, HL-SMDS 150) was observed (Figure S1, Supplementary Material). The average Systolic Blood Pressure SBP in the L-NAME-induced hypertensive rat group was significantly higher (158 mmHg) than that in the normotensive rat group. Figure 1 shows that all treatments induced a significant decrease in SBP compared with that of the L-NAME group. As expected, the antihypertensive effect of the lipid system was significantly greater than that of the crude extract at the same dose administered (150 mg/kg/day). Furthermore, the blood pressure values shown by the rats administered HL-SMDS were similar to those of the normotensive control group.

### 2.4. Effect of Oral Administration of HL-SMDS on the Vascular Reactivity: Ex Vivo Experiments

**Figure 2:**
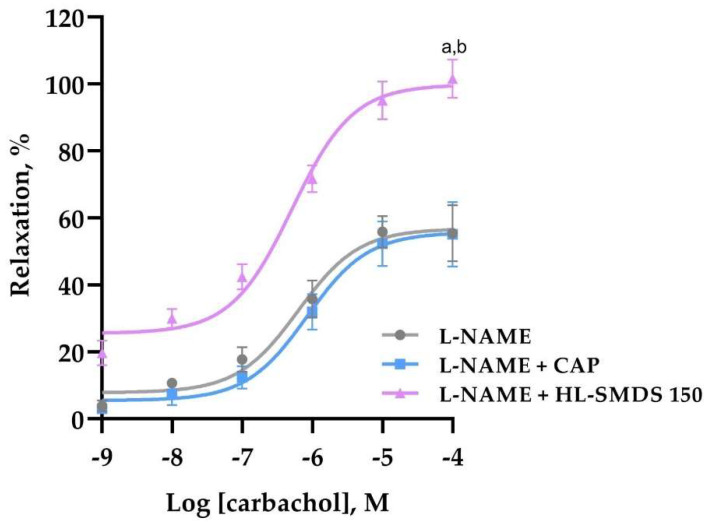
Effect of oral administration of HL-SMDS (150 mg/kg/day) and CAP (100 mg/kg/day) on the vascular reactivity after 4 days of treatment. Concentration–response curves are presented as the relaxation percentage of rat aortic rings pre-contracted by L-phenylephrine (1 μM) as a function of carbachol concentration. Values are shown as the mean ± SEM (*n* = 5 rats for each group). ^a^ *p* < 0.05 vs. L-NAME, ^b^ *p* < 0.05 vs. CAP.

The effect of oral administration of HL-SMDS on the vascular reactivity of isolated aortas obtained from L-NAME-induced hypertensive rats was determined. As shown in Figure 2, the carbachol-induced relaxation of aortas from rats of the L-NAME group and rats administered with captopril was significantly reduced by half. However, aortas from rats treated with HL-SMDS showed a significantly higher vasodilation that reached up to 100%. These findings suggest that HL-SMDS increased the endothelial reactivity.

### 2.5. Determination of the Antihypertensive Effect in Spontaneously Hypertensive Rats (SHRs)

**Figure 3:**
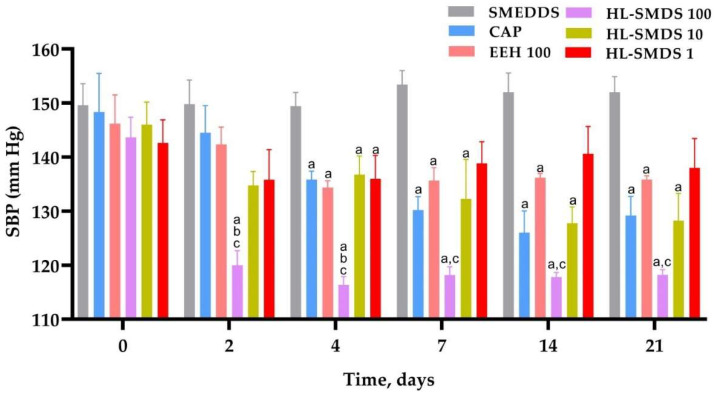
Effect of long-term oral administration of HL-SMDS (100, 10, and 1 mg/kg/day), EEH (100 mg/kg/day), and CAP (100 mg/kg/day) for 21 days on the SBP of SHRs. Doses of HL-SMDS corresponding to 1, 10, and 100 mg/kg contain approximate doses of affinin of 0.34, 3.4, and 34 mg/kg, respectively. Values are the mean ± SEM (*n* = 6 rats for each group). ^a^ *p* < 0.05 vs. SMEDDS; ^b^ *p* < 0.05 vs. CAP; ^c^ *p* < 0.05 EEH vs. HL-SMDS; using one-way ANOVA followed by Tukey’s post hoc test.

The initial SBP of the SHRs used in the present study was 146 ± 1.25 mmHg. Two days after starting the treatment, HL-SMDS 100 induced a significant decrease in SBP up to values of 120 ± 2.68 mmHg, which remained stable until the end of the experiment (118 ± 0.97 mmHg). The antihypertensive effect of HL-SMDS 100 was significantly greater than that of the self-microemulsifying drug delivery system (SMEDDS), CAP, and EEH 100 groups (Figure 3), whose hypotensive action started four days after beginning the administration of the treatments. Regarding HL-SMDS 10, it produced an antihypertensive effect, which was comparable to that of captopril, throughout the experiment. In the case of the HL-SMDS 1 group, the blood pressure of these rats decreased transiently and started to gradually increase until reaching the SBP values observed at the beginning of the experiment.

All doses of HL-SMDS (1, 10, and 100 mg/kg/day) induced a decrease in the SBP of SHRs in a dose-dependent manner (138, 128, and 118 mmHg, respectively). Throughout the experiment, significant differences were obtained between the antihypertensive effects of HL-SMDS 100 and EEH 100 (100 mg/kg/day), which may be attributed to the increased solubility of the quantified EEH in the SMEDDS. As in the case of the experiments carried out in the L-NAME-induced hypertensive rats, the HL-SMDS significantly increased the antihypertensive effect of the crude extract obtained from *H. longipes* root.

### 2.6. Effect of Long-Term Oral Administration of HL-SMDS on the Vascular Reactivity: Ex Vivo Experiments

**Figure 4:**
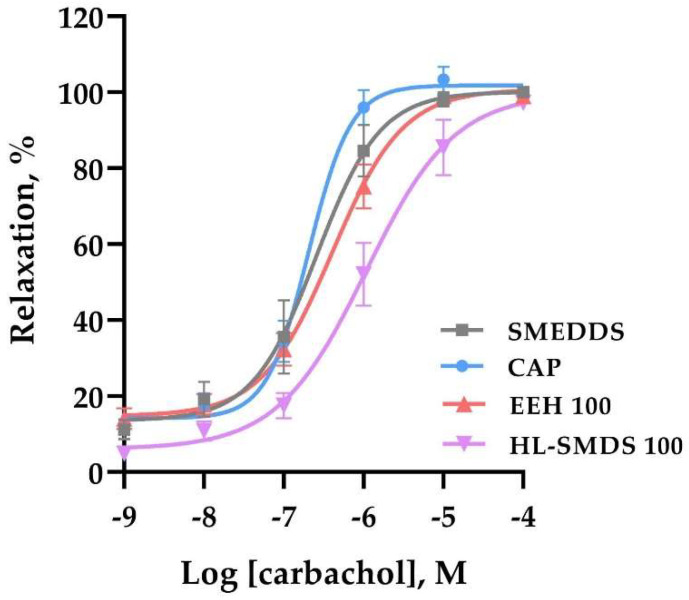
Effect of long-term oral administration of HL-SMDS 100 (100 mg/kg/day), EEH 100 (100 mg/kg/day), and CAP (100 mg/kg/day) on the vascular reactivity after 21 days of treatment. Concentration–response curves are presented as the percentage of relaxation of rat aortic rings pre-contracted by L-phenylephrine (1 μM) as a function of carbachol concentration. Values are shown as the mean ± SEM (*n* = 6 rats for each group). *p* < 0.05 vs. SMEDDS.

To determine if there was a decrease in endothelial reactivity once the antihypertensive experiment was completed, endothelial viability was assessed. The results obtained are shown in Figure 4. Unlike the experiment where hypertension was induced with L-NAME, in this experiment, 100% relaxation was obtained in the four groups evaluated. These results indicate that the SHRs did not exhibit endothelial dysfunction at the time that the experiment was conducted. It is important to mention that the aortas of these groups had greater coverage with fatty tissue, indicating the possible existence of hyperlipidemia. This was not observed in the aortas of hypertensive rats induced by L-NAME, which indeed exhibited endothelial dysfunction.

### 2.7. SHR Heart Analysis

Upon completion of the antihypertensive study, the hearts of the SHRs from all groups were analyzed, and the results are presented in Table 2. A relationship was observed between the SBP values of the SHRs and their heart dimensions; specifically, as the SBP values increased, the heart dimensions also increased. This correlation was confirmed through measurements of the heart wall thickness. Significant differences were observed between the untreated hypertensive control group and both the CAP and HL-SMDS 100 groups. Although no statistically significant differences were found in the heart index, a trend was noted, with the most pronounced difference being observed between the SMEDDS and HL-SMDS 1 groups.

### 2.8. Quantification of Nitric Oxide Production in Serum

**Figure 5:**
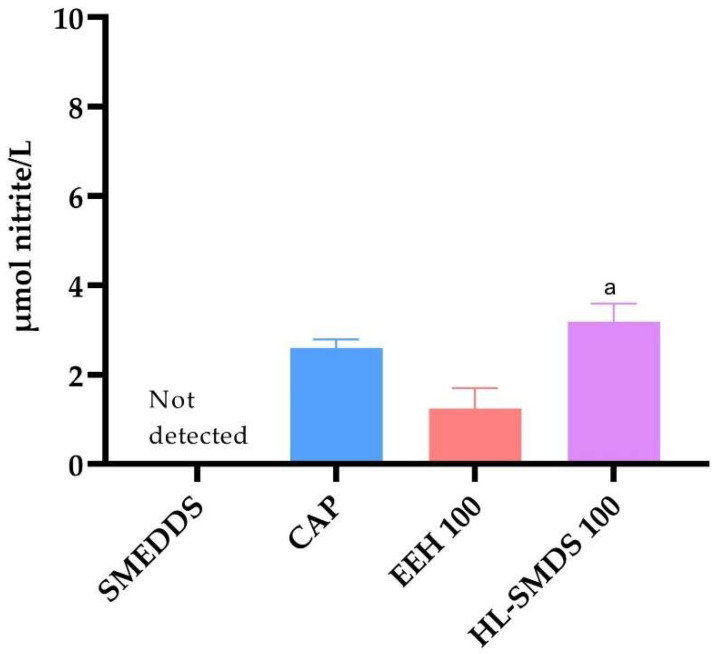
Serum nitrite levels in SHRs treated with HL-SMDS 100 (100 mg/kg/day), EEH 100 (100 mg/kg/day), and CAP (100 mg/kg/day). The values are expressed as the ±SEM (*n* = 4). ^a^ *p* < 0.05 vs. EEH 100.

Nitrite levels in the serum of SHRs treated with HL-SMDS were significantly higher than those in the group treated with EEH. In contrast, no significant differences were observed when compared to the positive control group (Captopril), and nitrite levels were undetectable in the negative control group (Figure 5).

## 3. Discussion

High blood pressure is one of the most critical and costly public health challenges worldwide and represents a major risk factor for the development of cardiovascular diseases. According to the World Health Organization (WHO), approximately 10.3 million people die each year from hypertension, and only one in five adults with the condition has it under control [21]. Therefore, the identification of new antihypertensive drugs with novel mechanisms of action is essential for improving blood pressure treatment.

Recently, our research group demonstrated that the ethanolic extract of *H. longipes* root (EEH) and its principal alkamide, affinin, exert vasodilatory and antihypertensive effects [11,16,17]. However, EEH has poor water solubility, which presents a challenge for its oral administration and formulation as an herbal medicine. This characteristic is directly related to its chemical composition, which includes a high proportion of lipophilic compounds. In particular, alkamides account for 45.57% of the extract, with affinin being the major constituent (45.08%). These molecules possess long hydrocarbon chains, which reduce their affinity for polar media. Additionally, the presence of other hydrophobic compounds, such as stigmasterol (0.37%), triterpene lup-20(29)-en-3-one (0.23%), and squalene, further contributes to the limited solubility of EEH in aqueous media.

This solubility issue is common in the development of new drugs; it is estimated that approximately 80% of drug candidates are poorly water-soluble, which results in low bioavailability [22]. To address this challenge, our research group developed a self-microemulsifying drug delivery system (SMEDDS) that significantly improved the solubility of EEH and remained stable over time under extreme conditions. Moreover, the system substantially enhanced the release of affinin, the major bioactive compound found in *H. longipes* root [18].

To evaluate whether the HL-SMDS system enhances the antihypertensive effect of the crude extract, two in vivo murine models were used: L-NAME-induced hypertensive rats and spontaneously hypertensive rats (SHRs). The L-NAME-induced hypertension model offers several advantages, including ease of implementation, high reproducibility, the development of systemic hypertension, evidence of target-organ damage, and reversibility of hypertension with various antihypertensive agents [20]. In contrast, the SHR model is widely used to demonstrate the antihypertensive effects of both approved drugs and novel molecules or plant extracts [23,24,25].

As expected, HL-SMDS reduced the SBP in both hypertension models, demonstrating a greater antihypertensive effect than the crude extract at doses of 150 and 100 mg/kg/day. This enhanced effect may be attributed to the increased solubility of both the extract and affinin, which ensures complete dose delivery. This improved bioavailability could explain the significant differences in blood pressure values observed between rats treated with the HL-SMDS formulation and those receiving the crude extract.

Oral administration of HL-SMDS at the highest evaluated dose (100 mg/kg/day) reduced the SBP in SHRs as early as the second day of treatment. Remarkably, the SBP values remained around 120 mmHg throughout the 21-day experimental period, which is considered normal for normotensive rats [26]. In contrast, the 10 mg/kg/day dose produced SBP values comparable to those observed with captopril at 50 mg/kg/day.

The enhanced antihypertensive effect of HL-SMDS can be attributed to its improved bioavailability. Upon contact with gastrointestinal fluids, the SMEDDS forms microemulsions—micellar colloidal systems that protect the active compounds from degradation and precipitation, thereby enhancing their absorption. This results in increased bioavailability and, consequently, an improved pharmacological effect [27,28,29].

In previous in vitro experiments, we demonstrated that affinin release from HL-SMDS was greater than from EEH in both acidic (pH 1) and neutral (pH 6.8) media. In a pH 1 medium, the cumulative dissolution rate of affinin in HL-SMDS exceeded 50% within approximately 15 min, whereas affinin release from EEH reached only 16% [18]. These findings suggest that HL-SMDS is an effective formulation for enhancing both the solubility and pharmacological activity of the extract derived from *H. longipes* root.

Our results also demonstrated that endothelium-dependent vasorelaxation in response to carbachol was significantly reduced in the aortas of L-NAME-induced hypertensive rats, indicating the presence of endothelial dysfunction. In contrast, the group treated with HL-SMDS exhibited vasorelaxation levels of up to 100%. The fact that the micro-emulsion containing *H. longipes* extract and affinin is able to reduce endothelial dysfunction induced by L-NAME-induced hypertension—unlike captopril, which did not improve endothelial function—can be attributed to the involvement of multiple pathways. The GC–MS analysis clearly demonstrated that affinin is the major component of the ethanolic extract of *H. longipes* root, accounting for approximately 45% of its content. Previous studies from our group have shown that affinin-induced vasodilation involves the activation of the NO/cGMP, CO/cGMP, H_2_S/KATP, and PGI_2_/cAMP signaling pathways [11], as well as the HNO–TRPA1–CGRP pathway and the blockade of L-type voltage-dependent calcium channels. In addition, affinin has been reported to modulate CB1 cannabinoid receptors and TRPA1 and TRPV1 channels [16]. We also demonstrated that both the ethanolic extract of H. longipes root and affinin exert antihypertensive effects in L-NAME-induced hypertensive rats and improve endothelial function, effects associated with increased serum NO levels. More recently, we confirmed that the antihypertensive action of affinin involves the activation of CB1 cannabinoid receptors and TRPA1 and TRPV1 channels [17]. Collectively, these findings provide strong evidence that affinin is the principal compound responsible for the antihypertensive effect produced by HL-SMDS. Consequently, the ability of the self-microemulsifying drug delivery system containing *H. longipes* extract (HL-SMDS) to attenuate L-NAME-induced endothelial dysfunction—unlike captopril, which failed to improve endothelial function—may be attributed to the involvement of multiple signaling pathways.

Pharmacokinetically, affinin is a lipophilic molecule (log P = 3.2) capable of crossing the blood–brain barrier, and it exhibits rapid absorption with a short half-life in rats (1.13 h) [30]. These properties support the conclusion that, once absorbed, affinin exerts a rapid systemic effect consistent with the early onset of action observed in our experiments. Moreover, the HL-SMDS formulation was specifically designed to enhance solubility and oral absorption, likely improving affinin bioavailability and thereby contributing to a faster and more pronounced antihypertensive effect.

Given that *H. longipes* and affinin have previously been reported to exert anti-inflammatory effects [15,31], the improvement in endothelial function observed with HL-SMDS treatment may also be partially attributed to inflammation reduction.

According to several authors, endothelial dysfunction is associated with decreased nitric oxide bioavailability [32,33,34], which promotes vasoconstriction and induces a proinflammatory and prothrombotic state, thereby increasing the risk of cardiovascular disease. Moreover, systemic inflammation contributes to oxidative stress within the vascular wall, generating free radicals that react with NO and further reduce its bioavailability, thereby exacerbating endothelial dysfunction [35,36]. In activated endothelial cells, the expression of proinflammatory cytokines, chemokines, enzymes, and adhesion molecules is substantially upregulated [37]. Therefore, it is evident that endothelial inflammation plays a central role in the pathogenesis of vascular dysfunction.

By contrast, SHRs did not exhibit a decrease in the endothelial response to carbachol. In this model, endothelial dysfunction is primarily associated with aging. In fact, age is a key factor influencing vascular function in both SHRs and normotensive rats, with reduced endothelium-dependent relaxation typically being observed in older animals (older than 25 weeks) [38]. At the time that the endothelial reactivity studies were conducted, the SHRs used were approximately 16 weeks old.

SHRs develop cardiac hypertrophy between four and twelve weeks of age as a consequence of sustained high blood pressure. Early initiation of antihypertensive therapy reduces blood pressure and exerts cardioprotective effects. In this study, the groups treated with captopril and HL-SMDS at 100 mg/kg/day showed a significant reduction in heart wall thickness. Previous studies have demonstrated that captopril reduces cardiovascular mortality, in part by decreasing cardiac and vascular hypertrophy, as well as cardiac and renal fibrosis [24,39]. In the case of HL-SMDS at 100 mg/kg/day, this potential cardioprotective effect deserves further investigation.

## 4. Materials and Methods

### 4.1. Materials

Reagents and solvents used in the present investigation were obtained from JT Baker^®^ (Phillisburg, NJ, USA). Lipid excipients (Transcutol^®^ HP and Labrasol^®^) were donated by Lyontec^®^ Chemicals, S. de R. L. de C.V. (Mexico) from Gattefossé^®^ (Paramus, NJ, USA). Tween^®^ 80, Propylene glycol^®^, and affinin standards were acquired from ChromaDex^®^ Standards (Rancho Los Ángeles, CA, USA). Reagents used for the pharmacological evaluations were obtained from Sigma-Aldrich^®^ (St. Louis, MO, USA).

### 4.2. Plant Material

*H. longipes* roots (Asteraceae) were collected in Peñamiller, Querétaro, Qro., México. The plant species was identified by M. Sc. José Alejandro Cabrera Luna (*H. longipes* vouchers J. E. Castro R.1. and R.2.) and deposited in the Jerzy Rzedowski Herbarium (QMEX), Faculty of Natural Sciences, Autonomous University of Querétaro, Querétaro, Qro., México.

### 4.3. Preparation of the Ethanolic Extract from H. Longipes Root (EEH) and Affinin Quantification

One kilogram of the dried, ground root was macerated in absolute ethanol in a 1:10 ratio (ground plant material: solvent) for one week. The plant material was filtered, and this process was repeated twice with a fresh solvent each time. The filtrates were combined, and the solvent was removed with a rotary evaporator (Heidolph^®^ VV 2000, Schönwalde-Glien, Germany).

Affinin quantification was carried out on a WATERS^®^ 600E HPLC (Waters 600 Associates, Milford, MA, USA) with a diode array detector (WATERS 2998), employing a ZORBAX^®^ ECLIPSE XDB-C8 column (4.5 × 150 mm, 5 µm particle size and 120 Å pore size) (Agilent Techologies, Inc., Santa Clara, CA, USA). An isocratic mobile phase consisting of acetonitrile and 1% acetic acid in water (1:1) was used at a flow rate of 1 mL/min. The injection volume was 20 µL, and the run time was 9 min. To determine the affinin content, a calibration curve was plotted using an affinin standard in a range of 15 to 90 µg/mL (15, 30, 45, 60, and 90 µg/mL). Two milligrams of EEH was weighed, dissolved in 1 mL of ethanol of absolute HPLC grade, sonicated for 15 min, and filtered through a nylon membrane with a pore size of 0.45 µm. Subsequently, a 100 µL aliquot was diluted to 1 mL with the mobile phase and then injected (in triplicate) into the HPLC [18].

### 4.4. GC-MS Analysis of EEH

EEH was analyzed using a gas chromatograph (Agilent Technologies Inc. 7809A, Agilent Techologies, Inc., Santa Clara, CA, USA) coupled to a quadrupole mass spectrometry detector (Agilent Technologies, Inc. 5975C, Agilent Techologies, Inc., Santa Clara, CA, USA). A DB-5MS capillary column (60 m × 250 µm, 0.25 µm film thickness) (Agilent Techologies, Inc., Santa Clara, CA, USA) was used as the stationary phase, and high-purity helium (99.99%) served as the carrier gas at a flow rate of 1 mL/min. The oven temperature was programmed to start at 40 °C (held for 10 min), then increased to 140 °C at a rate of 3 °C/min, and held for 10 min. Subsequently, the temperature was raised to 220 °C (held for 5 min), followed by a final ramp to 300 °C at 10 °C/min, where it was held for 10 min. Ionization was carried out by electron impact at 70 eV with a mass range of 33 to 600 amu. Compounds were identified by comparing the obtained mass spectra with those reported in the NIST Mass Spectra Library 2023^®^ database [40].

### 4.5. Preparation of the Self-Microemulsifying System

The self-microemulsion (HL-SMDS) loaded with the quantified EEH was prepared using Transcutol^®^ HP (solubilizer) 0.55 g, Labrasol^®^ (surfactant) 0.35 g, and (1:1) Tween^®^ 80/Propylene glycol^®^ (PG) 0.1 g. These excipients were blended until a homogeneous mixture was obtained. Subsequently, the quantified EEH was added, weighing 38 mg for a 150 mg/kg/day dose, 25 mg for a 100 mg/kg/day dose, 2.5 mg for a 10 mg/kg/day dose, and 0.25 mg for a 1 mg/kg/day dose (taking as a reference a rat that weighed 250 g). In all cases, the weighed extract was added to 1 mL of formulation [18].

### 4.6. Evaluation of the Antihypertensive Effect of HL-SMDS in L-NAME-Induced Hypertensive Rats

Adult male Wistar rats (6 weeks old; 190–210 g) were provided by the Institute of Neurobiology of the National Autonomous University of México, Campus Juriquilla, Querétaro, México. Experimental animals were housed in polycarbonate cages in groups of three rats per cage under controlled environmental conditions (21 ± 2 °C; 40–60% relative humidity) with a 12:12 h light–dark cycle. Rats had free access to water and standard laboratory chow. After a one-week acclimatization period, the rats were randomly divided into five groups, each consisting of six animals (*n* = 6). All experimental procedures were conducted in accordance with the Mexican Official Norm NOM-062-ZOO-1999 and the International Council for Laboratory Animal Science (ICLAS) guidelines. The protocol was reviewed and approved by the Bioethics Committee of the Faculty of Chemistry, Autonomous University of Querétaro, Mexico (approval number CBQ20/116).

For the induction of arterial hypertension, N-nitro-L-arginine methyl ester (L-NAME) (30 mg/kg/day) was administered over four weeks to the adult male Wistar rats (250–300 g). The treatments used were administered in all cases in the last 4 days of the study (Table 3). L-NAME and captopril were dissolved in water. The EEH was diluted in 1% carboxymethylcellulose aqueous solution. All drugs were administered orally via gavage (1 mL).

#### 4.6.1. Blood Pressure Measurement

To measure the systolic blood pressure (SBP), the tail-cuff method was used with a LE 5650/6 heater scanner (Panlab, Barcelona, Spain) coupled to an LE 5007 automatic blood pressure detector (Letica, Barcelona, Spain). Determinations were made weekly, and the mean value from at least five consecutive readings was used for calculations [17,41].

### 4.7. Effect of the Oral Administration of HL-SMDS on the Vascular Reactivity: Ex Vivo Experiments

At the end of the antihypertensive experiment, the thoracic aortas of the control groups (L-NAME and Captopril) and the group treated with HL-SMDS (HL-SMDS 150) were isolated to determine the effect of the lipid system treatment on the vascular reactivity using the isolated rat aorta assay. After equilibration, the aortic rings were contracted with 1 µM phenylephrine (PE). When a steady contraction was reached, the response to increasing concentrations of carbachol (CCh) (10^−9^–10^−4^ M) was tested. Relaxation was expressed as a percentage of the maximal PE-induced contraction [41].

### 4.8. Determination of the Antihypertensive Effect in Spontaneously Hypertensive Rats (SHRs)

Male spontaneously hypertensive rats (SHRs) (8–10 weeks old; 250–300 g) were purchased from the Institute of Cellular Physiology, National Autonomous University of Mexico. Throughout the experiment, animals were housed in polycarbonate rodent cages in groups of three per cage under controlled environmental conditions (21 ± 2 °C; 40–60% relative humidity) with a 12:12 h light–dark cycle. Rats had free access to water and standard laboratory chow. After a one-week acclimatization period, the rats were randomly divided into six groups, each consisting of six animals (*n* = 6). All experimental procedures were conducted in accordance with the Mexican Official Norm NOM-062-ZOO-1999 and the International Council for Laboratory Animal Science (ICLAS) guidelines. The protocol was reviewed and approved by the Bioethics Committee of the Faculty of Chemistry, Autonomous University of Querétaro, Mexico (approval number CBQ20/116).

The treatments were administered orally via gavage (1 mL) for 21 consecutive days in all cases (Table 4). To measure the SBP, the method described in Section 4.6.1 was employed.

### 4.9. Effect of the Oral Administration of HL-SMDS on the Vascular Reactivity: Ex Vivo Experiments

At the end of the antihypertensive experiment in SHR, the thoracic aortas of the SMEDDS, Captopril, EEH 100, and HL-SMDS 100 groups were isolated, and the methodology described in Section 4.7 was used.

### 4.10. SHR Heart Analysis

Upon completion of the antihypertensive experiment in SHRs, the hearts were excised, and their size was measured using a caliper. They were subsequently weighed, and their volume was obtained by displacing water in a test tube. Finally, a cut was made, and the thickness of the heart walls was measured.

The heart index (HI) was calculated using the following formula:

HI (%) = (Heart weight (g))/(Rat weight (g)) × 100

### 4.11. Quantification of Nitric Oxide in Serum

Blood samples were obtained from the SHRs (SMEDDS, Captopril, EEH 100, and HL-SMDS 100 groups); the serum was separated via centrifugation at 2500 rpm for 10 min (Sorvall SR70, Thermo Scientific, Inc., Waltham, MA, USA) and stored at −80 °C. Prior to analysis, samples were deproteinized using ultrafiltration with 10 kDa filters. The total amount of nitrites was measured using the Nitrate/Nitrite Colorimetric Assay Kit (Cayman Chemical, Item: 780001, Batch: 0708459, Ann Arbor, MI, USA) according to the instructions of the manufacturer.

### 4.12. Statistical Analysis

Data are reported as standard error of the mean ± SEM; they were analyzed using a one-way ANOVA test, and the differences between them were assessed using Tukey’s test. The differences between the means were considered significant when *p* < 0.05. Data analysis was performed with the GraphPad Prism 9.3 program.

## 5. Conclusions

The present study demonstrated that HL-SMDS significantly enhances the antihypertensive effect of the ethanolic extract of *H. longipes* root. This effect is associated with increased bioavailability of affinin, the principal vasodilator compound present in the crude extract. Moreover, HL-SMDS improved endothelial reactivity in aortas from rats with endothelial dysfunction. In addition, spontaneously hypertensive rats (SHRs) treated with the highest dose of HL-SMDS exhibited thinner heart walls. Collectively, these findings suggest that HL-SMDS may serve as a promising formulation for the development of an effective herbal medicine for the treatment of arterial hypertension.

## Figures and Tables

**Table 1:** Major volatile compounds identified in EEH.

RT	NAME	NO. CAS	MATCH	% AREA	TYPE
23.23	3-Methyl crotonic acid	000541-47-9	94	0.11	Fatty acid
28.64	α-Phellandrene	000099-83-2	87	0.02	Terpene (mono)
29.896	*p*-Cymenene	000099-87-6	97	0.02	Terpene (mono)
33.59	*o*-Cymene	007399-49-7	96	0.02	Terpene (mono)
39.07	α-Terpineol	010482-56-1	91	0.02	Terpene (mono)
41.06	Thymol methyl ether	001076-56-8	95	0.03	Terpene (mono)
45.15	*p*-vinylguaiacol	007786-61-0	80	0.04	Phenol
50.40	δ-Selinene	028624-23-9	86	0.06	Terpene (sesqui)
53.988	Isoeugenol	005932-68-3	95	0.07	Phenol
55.82	*n*-Nonyl-cyclopropane	074663-85-7	93	0.02	Alkane
75.71	Cyclododecane	000294-62-2	90	0.06	Cycloalkane
85.22	N-isobutyl-(6*Z*,8*E*)-decadienamide	094450-20-1	91	0.03	Alkamide
92.14	N-isobutyl-2(*E*),6(*Z*),8(*E*)-decatrienamide (affinin)	1000106-17-5	90	45.08	Alkamide
94.11	*cis*-9-Hexadecenoic acid	1000333-19-5	99	0.14	Fatty acid
97.51	N-(2-methylbutyl)-(2*E*,6*Z*,8*E*)-decatrienamide	100106-16-0	64	0.41	Alkamide
103.17	Linoleic acid	000060-33-3	90	0.09	Fatty acid
103.40	trans-13-Octadecenoic acid	000693-71-0	84	0.1	Fatty acid
124.65	Lup-20(29)-en-3-one	001617-70-5	91	0.23	Terpene (tri)
128.36	Taraxasterol	001059-14-9	95	0.21	Terpene (tri)
132.95	(*Z*)-13-Docosenamide	000112-84-5	91	0.04	Alkamide
135.10	Squalene	000111-02-4	98	0.11	Terpene (tri)
136.96	Eicosane	000112-95-8	89	0.02	Alkane
140.58	β-Tocopherol	000148-03-8	93	0.19	Phenol
140.91	γ-tocopherol	007616-22-0	93	0.09	Phenol
145.28	Dilauryl β,β-thiodipropionate	000123-28-4	90	0.19	Fatty acid
146.04	Campesterol	000474-62-4	81	0.05	Phytosterol
147.05	Stigmasterol	000083-48-7	99	0.37	Phytosterol

**Table 2:** Results obtained from the analysis of the hearts of SHRs.

Groups	Heart Size (Height/Width) (mm)	Heart Volume (mL)	Thickness of the Walls of the Heart (mm)	Heart Weight (g)	Heart Index (HW/RW) (%)
SMEDDS	18.00/14.60 ± 0.57	2.00 ± 0.16	5.20 ± 0.37	1.57 ± 0.05	0.55 ± 0.04
CAP	18.75/13.75 ± 0.63	1.17 ± 0.17 ^a^	2.92 ± 0.49 ^a^	1.37 ± 0.05	0.49 ± 0.02
EEH 100	17.58/13.83 ± 0.33	1.67 ± 0.17	3.75 ± 0.48	1.42 ± 0.09	0.54 ± 0.03
HL-SMDS 100	17.00/13.00 ± 0.56	1.13 ± 0.14 ^a^	3.00 ± 0.41 ^a^	1.24 ± 0.10 ^a^	0.47 ± 0.03
HL-SMDS 10	19.33/13.67 ± 0.67	1.83 ± 0.17	3.33 ± 0.33	1.50 ± 0.00	0.51 ± 0.01
HL-SMDS 1	19.75/14.25 ± 0.55	2.00 ± 0.20	3.25 ± 0.48	1.50 ± 0.12	0.56 ± 0.04

The values are the mean ± SEM of six rats of the same group. ^a^  *p* < 0.05 vs. SMEDDS. SMEDDS: hypertensive control; CAP: captopril (50 mg/kg/day); EEH 100: EEH (100 mg/kg/day); HL-SMDS 100: HL-SMDS (100 mg/kg/day); HL-SMDS 10: HL-SMDS (10 mg/kg/day); HL-SMDS 1: HL-SMDS (1 mg/kg/day).

**Table 3:** Treatments administered to the different groups of experimental rats.

Groups	Dose
Control (CTR, normotensive rats)	-
L-NAME (hypertensive control)	Vehicle (CMC 1%)
Captopril (CAP, positive control)	Captopril (100 mg/kg/day)
EEH 150	EEH (150 mg/kg/day)
HL-SMDS 150	HL-SMDS (150 mg/kg/day)

**Table 4:** Treatments administered to the different groups of experimental rats.

Groups	Dose
SMEDDS (hypertensive control)	SMEDDS (Transcutol^®^ HP: 0.55 g, Labrasol^®^: 0.35 g, (1:1) -Tween 80^®^/PG^®^: 0.1 g)
Captopril (CAP, positive control)	Captopril (50 mg/kg/day)
EEH 100	EEH (100 mg/kg/day)
HL-SMDS 100	HL-SMDS (100 mg/kg/day)
HL-SMDS 10	HL-SMDS (10 mg/kg/day)
HL-SMDS 1	HL-SMDS (1 mg/kg/day)

## Data Availability

Data are contained within the article.

## Supplementary Materials

The following supporting information can be downloaded at: https://www.mdpi.com/article/10.3390/molecules30183711/s1, Figure S1. Effect of oral administration of EEH and HL-SMDS (150 mg/kg/day) over 4 days on the SBP of L-NAME-induced hypertensive rats.

## Abbreviations

The following abbreviations are used in this manuscript:

ANOVA	Analysis of variance
CAP	Captopril
CB1	Cannabinoid receptor 1
CCh	Carbachol
CMC	Carboxymethyl cellulose
CTR	Control
EEH	Ethanolic extract from *H. longipes* root
HI	Heart index
HL-SMDS	SMEDDS containing the ethanolic extract of *H. longipes* root
HPLC	High-performance liquid chromatography
L-NAME	L-NG-nitroarginine methyl ester
NO	Nitric oxide
PE	Phenylephrine
SBP	Systolic blood pressure
SHR	Spontaneously hypertensive rat
SEM	Standard error of the mean
SMEDDS	Self-microemulsifying drug delivery systems
TRPA1	Transient receptor potential cation channel, subfamily A, member 1
TRPV1	Transient receptor potential cation channel, subfamily V, member 1
UV/VIS	Ultraviolet–visible
