# Supplementary material for: Antihypertensive Effect of a Self-Microemulsifying System Obtained from an Ethanolic Extract of Heliopsis longipes Root in Spontaneously and L-NAME-Induced Hypertensive Rats
Source: Molecules. 2025 Sep 12;30(18):3711. doi: 10.3390/molecules30183711 (PMC12472576; doi:10.3390/molecules30183711)
Supplement: Supplementary file 1 [file molecules-30-03711-s001.zip › molecules-3808204-supplementary.pdf]

Supplementary material

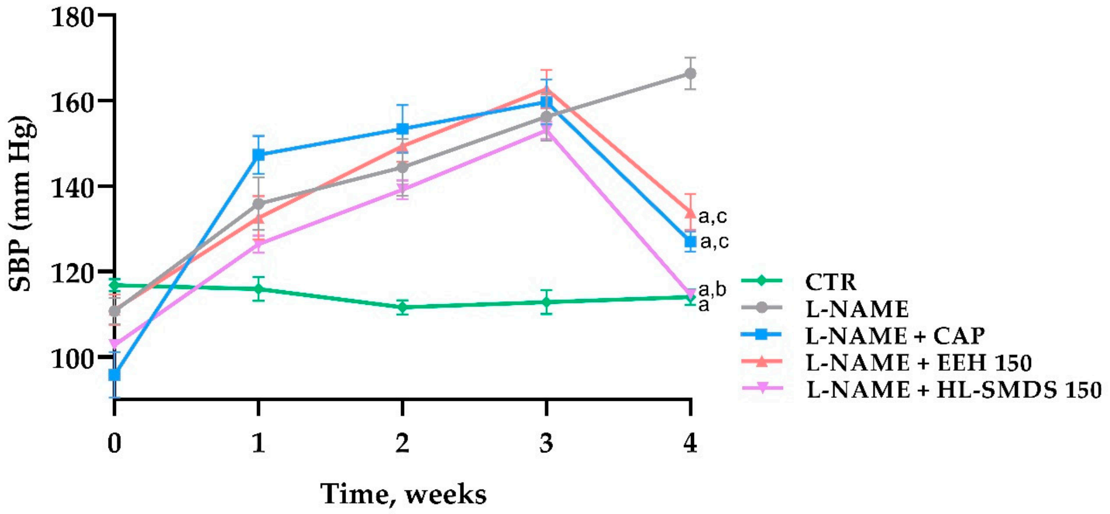

Figure S1. Effect of oral administration of EEH and HL-SMDS (150 mg/kg/day) during 4 days on the SBP of L- NAME-induced hypertensive rats. Captopril (CAP) (100 mg/kg/day) was used as positive control. Values are mean ± SEM (n = 6 rats for each group). a $p < 0.05$ vs L-NAME, b $p < 0.05$ EEH 150 vs HL-SMDS 150; c $p < 0.05$ vs CTR using one-way ANOVA followed by a Tukey's post hoc test
